# Child marriage and psychological well-being in Niger and Ethiopia

**DOI:** 10.1186/s12889-019-7314-z

**Published:** 2019-08-01

**Authors:** Neetu A. John, Jeffrey Edmeades, Lydia Murithi

**Affiliations:** International Center for Research on Women, 1120 20th Street, Suite 500 N, Washington, DC 20036 USA

**Keywords:** Child marriage, Psychological well-being, Mental health, Niger, Ethiopia

## Abstract

**Background:**

Despite an understanding of the circumstances of child marriage, including how it limits agency and erodes childhood support systems, not much is known about the relationship between child marriage and mental health of child brides, especially in the sub-Saharan African context. To address this gap, we use large-scale population-based data from ever-married women aged 18–45 in Niger (*n* = 2764) and Ethiopia (*n* = 4149) to examine the association of child marriage with overall psychological well-being and its sub-domains: depression, anxiety, positive well-being, vitality, self-control and general health. We complement this with qualitative data from Ethiopia to further contextualize the psychological well-being of child brides.

**Methods:**

Multivariate linear regressions were conducted to estimate the association between child marriage and overall psychological well-being and its sub-domains. Thematic qualitative analysis was conducted to further understand the lives of child brides.

**Results:**

Our regression analysis found significant negative associations between very early marriage (marriage at 15 years or earlier) and overall psychological well-being in both Niger and Ethiopia. With the exception of self-control, all sub-domains of psychological well-being – depression, anxiety, positive well-being, vitality and general health – were negatively associated with very early marriage. In addition, in the qualitative analysis, Ethiopian child brides reported suffering emotional distress and depression induced by the burden of handling marital responsibilities at an early age.

**Conclusion:**

The study highlights that even in settings where child marriage is normative, marrying very early is associated with negative outcomes. Further research is needed to understand the mechanisms that make those married during early adolescence particularly vulnerable to psychological distress, so that programs can address those vulnerabilities.

**Electronic supplementary material:**

The online version of this article (10.1186/s12889-019-7314-z) contains supplementary material, which is available to authorized users.

## Background

Child marriage continues to be highly prevalent in Africa, where almost 40% of girls are married before age 18 [1]. Research has consistently documented the adverse economic, social, demographic and reproductive health consequences of child marriage for child brides, their families and their communities [1–7]. Marriage can lead to unique changes in the life of an adolescent girl and requires transitioning abruptly into adult roles and responsibilities before she is developmentally ready to tackle these responsibilities [8]. In many parts of the world, for the bride, marriage also means moving to a new home and neighborhood and building new social networks which can exacerbate the mental health implications of child marriage [1]. Child marriage also limits the assets and resources available to girls by curtailing their education [1, 9], which results in diminished access to assets, resources and social support systems and limits the ability of married girls to exercise choice and agency in their lives [10, 11]. However, while researchers and programmers have long suggested that these changes may result in poorer mental health and psychological well-being, relatively little research has focused specifically on understanding the role of child marriage in this regard.

In the West, where a large body of literature on marriage, health and well-being exists, the evidence on the relationship between early marriage and mental health and overall psychological well-being is mixed. For example, one study from the United States utilizing population-based, nationally representative data among adults aged 18 years or older found that overall lifetime and 12 months’ rates of psychiatric disorders were higher among women who married as children than among those who married as adults [12]. However, another US-based study examining marriage and mental health among young adults, also using nationally representative data, found that while individuals who were married as teenagers reported more psychological distress compared to those who married later, the effects lost statistical significance after accounting for past psychological distress [13].

As opposed to the mixed evidence from the West, the limited evidence from low- and middle-income countries indicates a negative association between child marriage and mental health outcomes. For example, a study among adolescent Ethiopian girls from the Amhara region found increased odds of suicidality among girls who were ever married, were promised in marriage, or had received marriage requests when compared to unmarried girls [14]. Research from South Asia also suggests a higher risk for depression and suicidality among girls married as children, in particular due to increased exposure to gender based violence [15, 16]. However, in sub-Saharan Africa, several gaps remain in our understanding of the linkages between child marriage and mental health outcomes. For example, no study to date has examined the relationship between child marriage and psychological well-being in the general adult population. In addition, there is limited understanding of the factors that increase a child bride’s propensity for reduced psychological well-being and poor mental health outcomes.

To begin to address these gaps, we use unique large-scale population-based survey data from Niger and Ethiopia and examine the association between child marriage and psychological well-being among a sample of ever-married women aged 18–45 years. We limit our analysis to estimating the net association of child marriage on psychological well-being after accounting for other factors that may influence this relationship due to the cross-sectional nature of our data, which does not allow for a robust examination of pathways by which child marriage may impact psychological well-being. We try to address some of the limitations of the quantitative data with complementary qualitative data collected in Ethiopia from an urban and a rural site in Oromia and Amhara regions (Ethiopia’s first- and second-most populous regions respectively), which provides a more nuanced understanding of the kinds of psychological distress that child brides experience and the factors that promote these experiences overtime than is feasible using only cross-sectional survey data.

Despite both having relatively high rates of child marriage, the economic, cultural and social differences between Niger and Ethiopia provide a unique opportunity to understand how child marriage might influence psychological well-being. Niger has the highest rate of child marriage globally (over 75% of girls aged 15–19 are married), with prevalence having changed relatively little in the past two decades [17]. According to the 2012 Niger Demographic and Health Survey (DHS), 77% of women aged 25–49 were married by age 18, with a median age at first marriage of 15.7 (the lowest of all the countries for which DHS data are available) [18]. Ethiopia, too, has historically had very high rates of child marriage, particularly in areas such as Amhara, where the child marriage rate as recently as a decade ago rivaled that in Niger, though available data suggest a significant recent decline in prevalence [1]. According to the 2016 Ethiopia DHS data, 58% of women aged 25–49 were married by age 18, with a median age at first marriage of 17.1 [19]. Yet the two countries differ in a number of ways that provide an interesting contrast, including in religious traditions (Niger is predominantly Muslim while Ethiopia, while having a large Muslim population, is predominantly Orthodox Christian).

## Methods

### Data

The data used for these analyses were derived from a multi-country study intended to estimate the economic costs of child marriage. Ethical review at the global level was provided by the International Center for Research on Women’s IRB. In Ethiopia the review was conducted by the National Research Ethics Review Committee, while in Niger the review was provided by the Ministry of Public Health’s National Advisory Committee on Ethics. Survey data were collected in both countries from ever-married women in the age range of 18–45 years and male household heads using a multi-stage cluster design intended to generate broadly representative data for most of the country. In Niger, the survey was administered to 2764 women and their household heads in five of Niger’s eight regions – excepting Niamey, Agadez and Diffa. A two-stage stratified sampling approach was adopted to develop the sample. At first, the regions were stratified based on the number of households and within each region 200 enumeration areas (EA) were randomly selected, with 20 households randomly selected within each EA. In Ethiopia, 4149 women were interviewed in all nine regions and one town administration. A three-stage stratified sampling approach was used to develop the sample. First, within each region, districts were selected probability proportional to size, followed by random selection of counties and villages. Finally, 25 households were randomly recruited from each village. A household census was conducted in both countries for the random selection of eligible households. If a woman did not consent to participate, the field team selected another eligible woman from the same household or an adjacent household. The women’s questionnaire collected detailed information on the woman’s demographic background, health history (including mental health), relationship with her husband and experiences of intimate partner violence (IPV) (Please see Additional file 1). In addition, the household questionnaire collected information on household wealth, demographic and health information of the household members.

As noted above, in Ethiopia, the quantitative data were supplemented by qualitative data. Specifically, in-depth interviews (IDIs) were conducted with ever-married women aged 18–45, who were married before the age of 18 years, and had been married for at least five years. In addition, participatory focus group discussions (PFGDs) were held with mothers and fathers who had daughters between the ages of 8 and 17 years in an urban and a rural site in Oromia and Amhara regions. Study participants were purposively selected based on demographic, socio-economic and cultural characteristics of the population. A total of 32 IDIs (16 in each region) and 8 PFGDs (2 with fathers and 2 with mothers in each region) were completed. Of the 32 IDIs completed, half were with women aged 18–24 years and the other half with 25–45 year old women. Half of the 8 completed PFGDs were with women and the other half with men. The sample was equally split between the two study sites. These data were used to further explore topics including psychological well-being where the quantitative evidence was not sufficiently nuanced or to understand the temporal sequence and experiences of events in a child bride’s lives. These data provide an in-depth understanding of the lives of child brides, the normative context of their lives and the processes that may have influenced their experiences of psychological well-being over time.

### Quantitative measures

#### Key dependent variables

Psychological Well-Being: Measures of well-being were obtained from the Psychological General Well-being Index (PGWBI), a widely used scale to measure the psychological quality of life of general populations as well as people with chronic diseases [20]. The index consists of 22 items, which are rated on a 6-point scale to assess psychological well-being of respondents in 6 health-related quality of life domains: anxiety, depressed mood, positive well-being, self-control, general health and vitality. Internal consistency of the items was assessed using Cronbach’s Alpha. This was followed by principal components analysis (PCA), and the predicted scores generated from the PCA were normalized to range from 0 to 100, where a higher score indicates higher levels of well-being. In addition to the overall measure of psychological well-being, we also assessed psychometric properties of each of the sub-domains and created separate measures for each sub-scale with normalized scores ranging from 0 to 100 (see Appendix 1 for specific items that constitute the sub-scales).

#### Key independent variable

Age at first marriage: To examine the gradient of the effect of age at marriage, a series of dummy variables were created to represent women who were married at 12 years or earlier, at 13 years, at 14 years, at 15 years, at 16 years and at 17 years.

#### Other variables

Intimate Partner Violence (IPV): The IPV measures were drawn from the 2004 World Health Organization’s (WHO) Multi-Country Study on Women’s Health and Domestic Violence [21]. The survey includes measures of physical, sexual and emotional violence. Respondents were asked to report any IPV experienced in the 12 months preceding the survey as well as prior to the last 12 months. For this analysis, we created a binary variable to determine if the woman had experienced physical or sexual violence at any point in her life.

Community-level norms: To reduce bias caused by the broader social norms captured by individual level variables such as child marriage, measures of psychological well-being and IPV, we created community-level indicators for each of these variables. To create the index, the measures were aggregated from individual responses, excluding the index respondent’s response (using a leave-out mean approach).

In addition, a range of standard socio-demographic, household and couple-level variables known to influence psychological well-being and child marriage were included in the analysis such as the respondent’s age, education, number of children, household wealth, religion and spousal age-difference.

### Statistical analysis

A series of multivariate linear regression models were estimated using Stata 14 software and their adjusted coefficients and standard errors are presented to reflect strength of association and level of significance. We first estimated a model with the overall psychological well-being index as the dependent variable and child marriage as the key independent variable and then repeated the process with each sub-domain. The models were weighted for complex survey design, including clustering of data.

### Integration of qualitative findings

The qualitative data collection included IDIs and PFGDs. While the IDIs were designed to highlight the individual experiences of child brides, the PFGDs were intended to capture the broader social norms shaping child marriage and their impact on women. The IDIs elicited biographical narratives and tried to capture the lived experiences of child brides and participants’ perception of its impact on their lives particularly in terms of their life trajectory and their sense of well-being using a “life history” approach. The PFGDs captured normative perspectives of mothers and fathers with daughters aged 8–17 years and asked participants to reflect upon and map the life of a girl who marries early in their community (before age 18), compared to one who marries later.

The interviews were audio recorded, followed by verbatim transcription and translation into English. The transcripts were coded thematically using a structured coding scheme and NVivo 10 qualitative data analysis software after testing for inter-coder agreement on 10% of the transcripts. After completion of coding, the team summarized the key themes.

## Results

### Quantitative

The total sample included 2764 women in Niger and 4149 women in Ethiopia. However, after accounting for missing information, which included 4% of the sample not knowing their age at marriage, the final analytical sample in Niger was reduced to 2463 women. In Ethiopia, 12% of the sample did not know their age at first marriage, and this along with missing information on other variables left 3501 women in the analytical sample. On average, the women in the Niger sample were 29 years old, while the women in the Ethiopia sample were 30 years old (Table 1).

**Table 1:** Descriptive Statistics on Study Variables

Variables	% or Means (SD)	
	Niger (*n* = 2463)	Ethiopia (*n* = 3501)
Current Age	28.80 (7.33)	29.70 (7.44)
Schooling	13.39	35.77
Employed	3.15	14.29
No of Children	3.70 (2.37)	3.57 (2.13)
Polygamy	24.04	8.63
Spousal Age Difference	10.82 (7.10)	7.06 (5.56)
Intimate Partner Violence (IPV)	10.77	29.71
Overall Psychological Well-being	71.02 (22.02)	66.35 (22.73)
Anxiety (ANX)	76.59 (25.16)	71.65 (29.32)
Depression (DEP)	75.62 (27.27)	80.66 (23.09)
Self-Control (SC)	72.67 (23.95)	68.21 (27.37)
Vitality (VT)	65.52 (26.44)	57.83 (24.58)
Positive Well-being (PWB)	63.92 (20.39)	59.76 (20.80)
General Health (GH)	69.40 (28.09)	70.90 (27.50)
Married at 12 or before	5.24	6.78
Married at 13	5.95	3.69
Married at 14	13.75	8.65
Married at 15	28.89	17.77
Married at 16	14.5	12.70
Married at 17	12.32	12.56
Married at 18 or later	19.36	37.85

While 36% of the women from Ethiopia had attended school, only 13% of the Nigerien women had attended school. On average, the Ethiopian women (Mean: 3.57; SD: 2.13) and Nigerien women (Mean: 3.70; SD: 2.37) had the same number of children. As many as 5.24% of the Nigerien women were married by age 12 or earlier, and only 19.36% of the women remained unmarried by age 17. In Ethiopia, on the other hand, while a slightly larger proportion of the women (6.78%) were married by age 12 or earlier, a larger proportion (35.77%) remained unmarried by age 17.

For measures of psychological well-being, the women from Niger had a slightly higher overall sense of psychological well-being (Mean: 71.02; SD: 22.02) compared to the Ethiopian women (Mean: 66.35; SD: 22.73). Among the sub-domains, while the Nigerien women were less likely to be anxious (Mean: 76.59; SD: 25.16), the Ethiopian women were less likely to be depressed (Mean: 80.66; SD: 23.09). Furthermore, descriptive statistics suggest a monotonic relationship between child marriage and psychological well-being with psychological well-being increasing steadily as age at marriage increases, although this pattern appeared to be clearer in Niger than in Ethiopia (Table 2).

**Table 2:** Overall Psychological Well-being and sub-Scale Domain Scores by Age at Marriage in Niger and Ethiopia

Age at Marriage (in years)	≤ 12	13 Yrs.	14 Yrs.	15 Yrs.	16 Yrs.	17 Yrs.	≥18
Niger (*n* = 2463)							
Overall Psychological Well-being	66.20 (23.22)	67.96 (22.09)	70.25 (21.94)	71.24 (21.62)	72.29 (22.09)	71.16 (23.46)	71.61 (21.62)
Anxiety (ANX)	71.10 (26.82)	73.19 (27.06)	76.36 (25.05)	77.80 (24.48)	76.79 (25.41)	76.44 (26.64)	77.03 (24.59)
Depression (DEP)	70.59 (28.03)	72.44 (28.50)	75.28 (27.62)	76.42 (26.48)	76.70 (27.22)	75.29 (29.49)	75.61 (27.04)
Self-Control (SC)	70.57 (24.19)	70.13 (25.82)	72.17 (24.68)	71.77 (24.09)	74.16 (23.19)	72.31 (24.73)	73.90 (23.47)
Vitality (VT)	63.49 (26.49)	64.59 (26.78)	66.09 (26.90)	66.53 (26.41)	65.87 (26.69)	65.87 (26.92)	65.57 (25.92)
Positive Well-being (PWB)	59.03 (20.35)	61.54 (19.39)	62.66 (20.00)	62.81 (20.50)	66.95 (20.11)	65.17 (21.44)	64.55 (4.58)
General Health (GH)	63.57 (29.72)	64.83 (29.62)	68.42 (28.31)	69.72 (27.98)	70.31 (27.48)	69.2 (29.42)	70.43 (28.15)
Ethiopia (*n* = 3501)							
Overall Psychological Well-being	62.11 (24.91)	63.45 (24.23)	64.86 (20.92)	65.04 (22.48)	66.90 (22.62)	68.79 (23.06)	68.64 (21.71)
Anxiety (ANX)	67.24 (30.19)	68.46 (30.87)	70.08 (27.70)	70.19 (29.57)	72.85 (29.60)	75.40 (28.80)	74.17 (27.78)
Depression (DEP)	78.58 (23.35)	78.16 (22.49)	80.48 (21.28)	79.32 (25.05)	81.50 (22.88)	83.15 (22.96)	81.82 (22.54)
Self-Control (SC)	66.40 (29.39)	67.37 (25.98)	68.15 (24.98)	67.74 (26.91)	68.66 (27.01)	69.18 (27.90)	69.49 (27.37)
Vitality (VT)	49.70 (25.61)	53.95 (25.64)	55.82 (24.16)	56.81 (23.69)	58.30 (23.66)	60.20 (24.49)	60.44 (24.40)
Positive Well-being (PWB)	54.94 (21.86)	58.50 (21.58)	58.55 (22.38)	59.02 (20.77)	59.17 (20.05)	60.71 (20.51)	61.77 (20.22)
General Health (GH)	69.09 (29.04)	67.28 (28.78)	68.80 (28.08)	69.60 (29.12)	73.28 (27.35)	73.45 (28.07)	74.69 (25.79)

Results from the multivariate regression analysis indicate a statistically significant negative association between very early marriage (marriage at 15 or earlier) and overall psychological well-being (Tables 3 and 4). In addition, in both countries, all sub-domains of psychological well-being except 'self-control' were clearly negatively associated with child marriage, with the influence of individual covariates generally consistent with those observed for the overall measure (Tables 3 and 4). In Niger, being married at any age up to age 15 had a statistically significant negative association with overall psychological well-being, with the largest association (relative to those married at 18 or older) for those married at age 12 or earlier (a reduced overall psychological well-being by 7.41 percentage points (β = − 7.41, SE: 2.26) and growing smaller as age at marriage increased: being married at 15 resulting in a reduced overall psychological well-being by 3.27 percentage points (β = − 3.27, SE: 1.59). In Ethiopia, a statistically significant relationship was only observed for those who married at age 12 or earlier; marriage before 12 years reduced overall psychological well-being by 5.09 points (β = − 5.09, SE: 2.03) compared to women who were married at 18 years or later. Among other variables, IPV was strongly negatively associated with psychological well-being in both countries. While having more children, especially up to 5, was positively associated with psychological well-being in Niger, the pattern was less clear in Ethiopia. On the other hand, in Ethiopia, being wealthier was clearly statistically significantly associated with increased psychological well-being, while in Niger this pattern was evident only when comparing the poorest with the poor.

**Table 3:** Results from the Multivariate Linear Regressions Analysis Examining the Association of Child Marriage (CM) on Overall Psychological Well-being (O-PWB) and its Sub-Domains in Niger (*n* = 2463)

VARIABLES	Overall^a^	ANX^a^	DEP^a^	SC^a^	VIT^a^	PWB^a^	GH^a^
Married at 12 or Earlier	−7.14	−9.42	−8.26	− 1.92	−8.90	−6.19	−8.18
	(2.26)**	(2.73)***	(2.98)**	(2.68)	(2.83)**	(1.87)**	(3.14)**
Married at 13	−5.06	−5.46	−4.40	−4.52	−4.54	−3.82	−7.19
	(2.06)**	(2.59)**	(2.85)	(2.35)*	(2.36)*	(1.69)**	(2.86)**
Married at 14	−2.90	−3.24	− 3.83	−0.75	− 3.25	−1.81	− 3.80
	(1.59)*	(1.84)*	(2.34)	(1.80)	(2.04)	(1.33)	(2.24)*
Married at 15	−3.27	−2.85	− 3.14	− 3.07	−3.02	− 3.09	−4.21
	(1.59)**	(1.82)	(2.07)	(1.83)*	(1.95)	(1.34)**	(2.12)**
Married at 16	−2.98	−3.59	−3.49	− 2.35	− 3.04	−1.28	−4.03
	(1.87)	(2.27)	(2.49)	(1.92)	(2.21)	(1.29)	(2.61)
Married at 17	−3.08	−3.55	−3.89	− 3.26	−2.25	−1.39	−3.63
	(1.90)	(2.06)*	(2.55)	(2.04)	(2.35)	(1.51)	(2.37)
Current Age	−0.14	−0.10	−0.21	− 0.08	− 0.19	− 0.06	− 0.25
	(0.09)	(0.11)	(0.11)*	(0.10)	(0.11)*	(0.08)	(0.12)**
Schooling	2.63	3.10	2.59	2.62	2.22	2.71	2.61
	(1.61)	(1.75)*	(2.05)	(1.68)	(1.99)	(1.42)*	(2.34)
Employed	−1.17	0.58	0.68	−3.24	−3.32	−0.49	− 1.70
	(2.92)	(3.41)	(3.31)	(3.06)	(3.62)	(2.64)	(4.26)
*1–2 Children*	4.10	5.47	4.98	6.55	2.86	2.58	2.07
	(1.98)**	(2.38)**	(2.49)**	(2.31)**	(2.50)	(2.17)	(2.58)
*3–5 Children*	5.50	7.23	6.89	7.14	4.56	2.46	4.67
	(1.91)**	(2.36)**	(2.27)**	(2.16)**	(2.41)*	(1.98)	(2.52)*
*6 or More*	3.04	3.91	4.27	4.48	1.64	0.76	2.51
	(2.22)	(2.71)	(2.74)	(2.40)*	(2.81)	(2.11)	(3.12)
IPV	−12.47	−14.18	−12.55	−12.83	−15.02	−9.85	− 11.34
	(2.18)***	(2.42)***	(2.75)***	(2.34)***	(2.84)***	(1.94)***	(2.45)***
Polygamy	0.29	0.83	0.99	0.09	−0.36	−1.09	0.93
	(1.15)	(1.40)	(1.53)	(1.21)	(1.36)	(0.96)	(1.48)
Spousal Age Difference							
*6–10 years*	0.91	1.55	1.52	0.35	2.21	0.54	−0.35
	(1.24)	(1.45)	(1.51)	(1.43)	(1.48)	(1.08)	(1.64)
*10 and More*	0.22	−0.23	−0.03	0.08	2.68	0.32	−0.04
	(1.29)	(1.57)	(1.59)	(1.32)	(1.65)	(1.11)	(1.69)
*Poorer House*	−3.09	−2.66	−3.41	−4.31	−2.72	−3.16	−2.74
	(1.45)**	(1.91)	(1.97)*	(1.55)**	(1.70)	(1.22)**	(1.76)
*Poor Household*	−1.18	−0.93	−0.78	−1.47	−1.07	−1.76	−1.38
	(1.30)	(1.61)	(1.81)	(1.54)	(1.71)	(1.18)	(1.73)
*Richer Household*	−1.32	− 1.28	− 1.44	−2.04	− 1.11	−0.69	−1.73
	(1.59)	(1.91)	(2.05)	(1.68)	(2.01)	(1.43)	(1.94)
*Richest Household*	−1.07	−0.23	−0.73	− 1.65	− 1.14	−1.26	− 1.44
	(1.67)	(2.04)	(2.10)	(1.81)	(2.08)	(1.60)	(2.01)
Community (O-PWB)	0.74	0.72	0.75	0.65	0.69	0.71	0.73
	(0.03)***	(0.04)***	(0.03)***	(0.04)***	(0.04)***	(0.03)***	(0.04)***
Community (CM)	−0.27	− 0.47	− 0.53	0.14	− 0.43	− 0.24	− 0.13
	(0.32)	(0.40)	(0.43)	(0.42)	(0.40)	(0.30)	(0.42)
Community (IPV)	6.23	6.02	7.66	4.43	4.71	4.83	5.13
	(2.96)**	(3.54)*	(3.75)**	(3.28)	(4.05)	(2.67)*	(4.06)

Standard errors in parentheses

*** *p* < 0.001, ** *p* < 0.05, * *p* < 0.1

^a^Models also adjusted for regions and urban/rural residence, and weighted for survey design

**Table 4:** Results from Multivariate Linear Regression Analysis Examining the Association of Child Marriage (CM) on Overall Psychological Well-being (O-PWB) and its Sub-Domains in Ethiopia (*n* = 3501)

VARIABLES	Overall^a^	ANX^a^	DEP^a^	SC^a^	VT^a^	PWB^a^	GH^a^
Married at 12 or Earlier	−5.09	−5.34	−3.75	−1.92	−6.63	−5.63	−5.13
	(2.03)**	(2.55)**	(1.80)**	(2.38)	(2.08)**	(1.70)**	(2.31)**
Married at 13	−2.58	−2.27	−3.34	0.60	−2.66	−1.16	−5.28
	(1.70)	(2.17)	(1.76)*	(1.77)	(2.09)	(1.58)	(2.18)**
Married at 14	−1.49	−1.52	−0.86	0.63	−1.44	−0.70	−4.14
	(1.50)	(1.91)	(1.34)	(1.76)	(1.72)	(1.47)	(1.90)**
Married at 15	−0.40	− 0.64	− 0.81	2.04	−0.31	− 0.11	−1.93
	(1.17)	(1.48)	(1.17)	(1.45)	(1.28)	(1.07)	(1.42)
Married at 16	−0.85	−0.84	−0.30	0.58	−0.97	− 0.99	−1.16
	(1.10)	(1.44)	(1.12)	(1.35)	(1.22)	(0.99)	(1.39)
Married at 17	0.67	1.56	1.04	0.34	0.40	0.09	−0.91
	(1.09)	(1.41)	(1.17)	(1.42)	(1.20)	(1.03)	(1.43)
Current Age	−0.41	−0.42	− 0.35	− 0.31	− 0.49	−0.24	− 0.50
	(0.07)***	(0.08)***	(0.07)***	(0.07)***	(0.09)***	(0.07)***	(0.09)***
Schooling	1.22	1.73	0.13	0.54	2.00	1.72	0.46
	(0.92)	(1.18)	(0.85)	(1.07)	(0.97)**	(0.83)**	(1.21)
Employed	−3.60	−6.08	−2.68	−0.01	−2.35	− 3.02	−4.59
	(1.41)**	(1.67)***	(1.38)*	(1.66)	(1.59)	(1.44)**	(1.66)**
*1–2 Children*	1.88	2.87	2.64	1.43	0.27	−0.11	2.98
	(1.18)	(1.47)*	(1.28)**	(1.46)	(1.44)	(1.23)	(1.52)*
*3–5 Children*	2.99	4.44	4.76	1.29	0.82	−0.12	4.67
	(1.31)**	(1.70)**	(1.49)**	(1.53)	(1.72)	(1.43)	(1.66)**
*6 or More*	3.07	3.81	6.67	1.94	0.81	−0.30	4.63
	(1.66)*	(2.19)*	(1.72)***	(2.05)	(2.01)	(1.66)	(2.22)**
IPV	−14.04	−17.27	−12.18	−11.55	−12.22	−10.90	−14.43
	(1.17)***	(1.60)***	(1.18)***	(1.31)***	(1.22)***	(1.15)***	(1.25)***
Polygamy	−2.34	−3.15	−2.21	−0.47	−1.87	−4.81	− 1.62
	(1.38)*	(1.79)*	(1.45)	(1.47)	(1.51)	(1.32)***	(1.59)
Spousal Age Difference							
*6–10 years age*	−1.05	−1.83	−1.08	− 1.50	−0.11	−0.55	−0.38
	(0.70)	(0.92)**	(0.81)	(0.83)*	(0.79)	(0.66)	(0.83)
*10 and More*	−1.88	−3.11	−1.24	−2.35	−0.92	− 1.63	−1.28
	(0.97)*	(1.26)**	(1.02)	(1.03)**	(1.25)	(0.99)	(1.18)
	3.83	5.49	1.89	3.81	5.28	2.45	2.26
	(1.29)**	(1.65)**	(1.15)	(1.55)**	(1.36)***	(1.15)**	(1.70)
*Poor* Household	2.95	3.95	1.18	4.07	3.06	1.88	1.91
	(1.12)**	(1.49)**	(1.10)	(1.43)**	(1.28)**	(1.06)*	(1.49)
*Middle-income house*	4.00	5.52	2.40	4.65	3.62	2.53	2.73
	(1.14)***	(1.52)***	(1.18)**	(1.42)**	(1.25)**	(1.06)**	(1.43)*
*Richer Household*	4.32	6.03	1.83	4.30	4.08	2.88	3.51
	(1.07)***	(1.46)***	(1.21)	(1.30)**	(1.29)**	(1.20)**	(1.36)**
*Richest*	5.45	7.09	2.79	5.78	5.31	0.47	2.79
	(1.17)***	(1.62)***	(1.36)**	(1.39)***	(1.38)***	(0.06)***	(1.32)**
Community (O-PWB)	0.69	0.65	0.65	0.77	0.56	0.57	0.51
	(0.04)***	(0.05)***	(0.05)***	(0.04)***	(0.05)***	(8.17)***	(0.06)***
Community (CM)	−0.07	−0.06	−0.06	0.30	0.03	−0.20	−0.31
	(0.25)	(0.31)	(0.21)	(0.27)	(0.28)	(0.24)	(0.32)
Community (IPV)	5.11	4.00	0.75	3.77	5.49	7.63	7.75
	(2.18)**	(2.71)	(2.15)	(2.45)	(2.46)**	(2.86)**	(2.17)***

Standard errors in parentheses

*** *p* < 0.001, ** *p* < 0.05, * *p* < 0.1

^a^Models also adjusted for regions and urban/rural residence, and weighted for survey design

### Qualitative

The qualitative data provide some additional insights into that ways in which child marriage impacted the mental health of child brides in Ethiopia. In particular, women reported that being forced into marriage, often to a stranger, and the burden of marital responsibilities, most notably partner’s sexual demands and childbearing and child-rearing, led to significant emotional distress and depression. In response to a question on how early forced marriage had affected her psychological well-being, one woman stated, “*I was depressed and cried all the time… How do you think it feels to be forced into a marriage and a life with someone you didn’t choose or know?”* Another woman who was married at the age of 14 years narrated her experience of physical and sexual assault by husband, *“It used to be so painful for me when we had intercourse. But I couldn’t tell that to anyone. And when I refused he used to beat me, splash water on me, put a rock on me and he waited till I got tired and took me afterwards… I wished I was dead.”* Asked how different her life would have been had she not married early, one woman lamented: *“I would be mature in making my own choices and I would not suffer in raising a child in my childhood. And you know what happened last year, it was my daughter’s birthday and I didn’t have any money to celebrate her birthday... This would not have happened if I was married years later and I would use family planning if I knew there was such a thing. I advise the government or NGOs to teach people about family planning to avoid unplanned pregnancy.”*

The reports in the IDIs of significant mental health implications as the result of marrying as children were generally supported by the PFGD participants, with the majority expressing that child brides were more likely than those who marry later to be stressed and anxious and be in unfulfilling marriages because of their youth and lack of readiness to tackle adult responsibilities, which is often further exacerbated by their reduced agency due to diminished educational and economic opportunities.

## Discussion

Our study found significant negative associations between very early marriage (marriage at 15 years or earlier) and overall psychological well-being in both Niger and Ethiopia. Other than ‘self-control,’ all sub-domains of psychological well-being – depression, anxiety, positive well-being, vitality and general health – were negatively associated with very early marriage. These findings remained strong even after adjusting for the prevalence of child marriage and violence and average psychological well-being at the community level, which suggests that even after accounting for some of the social norms captured by the child marriage variable and other community variables, very early marriage is detrimental to psychological well-being. Among other factors, IPV was strongly negatively associated with psychological well-being in both countries.

Several mechanisms may be at play in the relationship between early marriage and psychological well-being. Current research suggests that early marriage often exposes women to elevated risk of IPV, reduced communication with the husband/partner, lack of knowledge about controlling fertility, limited decision-making power and less access to resources compared to women who marry as adults [8, 16, 22–24]. Our qualitative data from Ethiopia also suggest that factors such as IPV, diminished decision-making ability and reduced access to financial and social resources are major factors that affect the psychological well-being of child brides.

This study has several limitations that need discussion to better interpret the results. The key measures, age at marriage and psychological well-being, were self-reported and therefore subject to recall and social desirability bias. To minimize these errors privacy was ensured, and examples such as historical events or seasons were used to support recall of age and other events. In addition, especially in Ethiopia, over 10% of the sample had missing information for age at marriage and could not be included in the analytical sample, and many of these excluded participants were poorer, less educated and more likely to be married as children, possibly biasing the results towards more favorable mental health outcomes. Also, given the cross-sectional nature of the survey, it is hard to fully account for endogeneity bias. However, given the normative preference for early marriage in both countries, the likelihood for psychological well-being to be a cause of early marriage seems very low. Moreover, the regressions controlled for many factors associated with child marriage and/or psychological well-being, including several community level variables. The cross-sectional nature of the data also limited our ability to examine the mechanisms by which child marriage impacts psychological well-being. Therefore, we were not able to parse out the direct and indirect effect of child marriage on psychological well-being, and hence the parameter estimates are likely to be an underestimation of the relationship between child marriage and psychological well-being. We tried to address this limitation partially by collecting nuanced qualitative data that could provide insights into the pathways by which child marriage influences psychological well-being. A longitudinal design would also have allowed us to explore the role that the women’s birth family and household played in shaping longer term psychological outcomes, further isolating the effect of child marriage specifically. We also were not able to explore what role a girl’s level of involvement in the marriage decision has in shaping longer term psychological effects in our analyses. Finally, our qualitative data are limited only to women who had married as children and comes from only two sites in Ethiopia.

Despite these limitations, our study provides a unique contribution to the literature by examining the link between child marriage and psychological well-being in such diverse contexts. To our knowledge, no other study has investigated this relationship using large scale population-based data, which were complemented by qualitative data in one context. Moreover, our survey collected detailed measures on psychological well-being and IPV which are not routinely collected by national level surveys. Finally, our study used exact age at marriage to measure child marriage, which allowed for a more nuanced understanding of the gradient of risk given the rapid physical and sexual changes, and brain development that characterizes the period of adolescence. Dixon-Mueller (2008) in their pioneering study “how young is too young,” after reviewing data on physiological and cognitive readiness and on common legal and international standards related to consent, concluded that while early adolescence (before age 15) is generally “too early” to start having sex or to get married, late adolescence (18–19 years) is probably “old enough” [25]. Our study found similar results; only the very young child brides or those who married in the period of early adolescence in Niger and Ethiopia were likely to have diminished overall psychological well-being as compared to women who married as adults, highlighting that even in settings where child marriage is normative, marrying very early is fraught with negative outcomes. More research is needed to understand the mechanisms that make those married during early adolescence particularly vulnerable to psychological distress.

## Conclusion

This study highlights that even in settings where child marriage is normative, marrying very early is fraught with negative outcomes. More research is needed to both understand the mechanisms that make those married during early adolescence particularly vulnerable to psychological distress as well as explore how best to mitigate the specific vulnerabilities girls have towards child marriage as well as poor mental health outcomes, so that programs can address those vulnerabilities. It is critical that more of this research use a longitudinal approach, which would allow for a full examination of the complexity of the determinants of both marriage and mental health. Finally, research is needed to explore the degree to which psychological distress may be related to the way the marriage was initiated and the level of engagement the girl herself had in the marriage decision.

## Additional file

Additional file 1: Women’s Questionnaire. (PDF 247 kb)

## Data Availability

Data are currently available upon request from ICRW and individuals can contact the lead author (njohn@icrw.org) to request data. The data will be made publicly available soon.

## Appendix 1

**Table 5:** Description of Items from the Psychological Well-Being Index (PGWBI)

S.NO	Psychological Well-Being Index Items
Anxiety (ANX)	
1	Have you been bothered by nervousness or your “nerves” during the past month?
2	Were you generally tense or did you feel any tension this past month?
3	Have you been anxious, worried or upset during the past months?
4	Did you feel relaxed, at ease or high strung, tight or keyed-up during the past month?
5	Have you been under or felt your were under strain, stress or pressure during the past month?
Depression (DEP)	
6	Did you feel depressed this past month?
7	I felt downhearted and blue this past month?
8	Have you felt so sad, discouraged, hopeless or had so many problems that you wondered if anything was worthwhile during this past month?
Self-Control (SC)	
9	Have you been in firm control of your behavior, thoughts, emotions or feeling during the past month?
10	Have you had any reason to wonder if you were losing your mind, or losing control over the way you act, talk, think, feel or your memory during the past month?
11	I was emotionally stable and sure of myself this past month
Vitality (VIT)	
12	How much energy, pep, or vitality did you have or feel during the past month?
13	I woke up feeling fresh and rested this past month?
14.	Did you feel active, vigorous, or dull, sluggish during the past month?
15.	I felt tired, worn out, used up, or exhausted during the past month
Positive Well-being (PWB)	
16.	How have you been feeling in general this past month?
17.	How happy, satisfied or pleased have you been with your personal life during the past month?
18.	My daily life was full of things that were interesting to me during the past month.
19.	I felt cheerful, lighthearted during the past month.
General Health (GH)	
20.	How often were you bothered by any illness, bodily disorder, aches or pains?
21.	Did you feel healthy enough to carry out the things you like to do or had to or
22.	Have you been concerned, worried, or had any fears about your health during the past month?

## Abbreviations

DHS: Demographic and Health Surveys
EA: Enumeration Areas
IDI: In-depth Interviews
IPV: Intimate Partner Violence
PCA: Principal Components Analysis
PFGD: Participatory Focus Group Discussion
PGWBI: Psychological General Well-being Index
SD: Standard Deviation
SE: Standard Error
WHO: World Health Organization
