# Supplementary material for: Child marriage and psychological well-being in Niger and Ethiopia
Source: BMC Public Health. 2019 Aug 1;19:1029. doi: 10.1186/s12889-019-7314-z (PMC6670185; doi:10.1186/s12889-019-7314-z)
Supplement: Supplementary file 1 — Women’s Questionnaire. (PDF 247 kb) [file 12889_2019_7314_MOESM1_ESM.pdf]

Economic Cost of Child Marriage Women's Questionnaire

Section 1. Respondent's background			
No	QUESTIONS AND FILTERS	CODING CATEGORIES	SKIP
101	In what month and year were you born?	Month ☐☐<br><br>Don't know month…………………98<br><br>Year ☐☐☐☐<br>Don't know year……………………9998	
102	How old were you at your last birthday?<br>COMPARE AND CORRECT 1XX AND/OR 1XX IF INCONSISTENT	AGE IN COMPLETED YEARS<br><br>______	
103	Have you ever attended school?	YES…………………..1<br>NO……………….....2	
104	What is the highest level of school you attended: primary, secondary, or higher?	Pre-school ……………………………..0<br>PRIMARY……………………………....1<br>SECONDARY…………………………….2<br>HIGHER…………………………………..3<br>Informal Education……………………….4	
105	What is the highest [GRADE/FORM/YEAR] you completed at that level?<br><br>IF COMPLETED LESS THAN ONE YEAR AT THAT LEVEL, RECORD '00'.	0. Did not finish any schooling<br>1. 1st grade<br>2. 2nd grade<br>3. 3rd grade<br>4. 4th grade<br>5. 5th grade<br>6. 6th grade<br>7. 7th grade<br>8. 8th grade<br>9. 9th grade<br>10. 10th grade<br>11. 11th grade<br>12. 12th grade<br>13. Didn't finish higher education<br>14. Finished higher education other than university<br>15. Didn't finish university<br>16. Graduated from a university<br>17. Adult education<br>18. Other basic education<br>19. Other church/mosque education<br>30.Other	
106	Check the respondent level (primary, secondary or higher)<br>Pre-school ……………………………..0<br>PRIMARY……………………………....1<br>SECONDARY…………………………….2<br>HIGHER…………………………………..3→109<br>Informal Education……………………….4		→109

107	Now I would like you to read this sentence to me:<br><br>SHOW CARD TO RESPONDENT<br><br>IF RESPONDENT CANNOT READ WHOLE SENTENCE,<br><br>Probe: can you read any part of sentence for me?	CANNOT READ AT ALL………………..1<br>ABLE TO READ PART OF THE SENTENCE………………………………2<br>ABLE TO READ THE WHOLE SENTENCE………………………………3<br>BLIND/VISUALLY IMPAIRED…………4	
108	Is this the household you lived in as a child, or have you moved to live here?	1.YES<br>2.NO	→122
Characteristics of natal household and childhood experiences			
Now I would like to ask some questions about your natal family and the house you grew up in. If this is that household, then please provide information for this household.<br><br>CHECK THAT THE RESPONDENT IS NOT LIVING IN THE SAME HOUSEHOLD AS HER PARENTS OR THE SAME HOUSEHOLD THAT SHE GREW UP IN (IF PARENTS HAVE DIED).			
109	Did your mother ever attend school?	YES……………………………………………1<br>NO……………………………………………..2<br>DON'T KNOW………………………………98	 →111<br>→111
110	What is the highest level of school your mother completed:<br>primary, secondary, technical/vocational or higher?	0. Did not finish any schooling<br>1. 1st grade<br>2. 2nd grade<br>3. 3rd grade<br>4. 4th grade<br>5. 5th grade<br>6. 6th grade<br>7. 7th grade<br>8. 8th grade<br>9. 9th grade<br>10. 10th grade<br>11. 11th grade<br>12. 12th grade<br>13. Didn't finish higher education<br>14. Finished higher education other than university<br>15. Didn't finish university<br>16. Graduated from a university<br>17. Adult education<br>18. Other basic education<br>19. Other church/mosque education<br>30.Other	
111	Did your father ever attend school?	YES…………………………………………..1<br>NO………………………………………...2<br>DON'T KNOW…………………………..98	 →113<br>→113

112	What is the highest level of school your father completed:<br>primary, secondary, technical/vocational or higher?	0. Did not finish any schooling<br>1.1st grade<br>1. 2nd grade<br>2. 3rd grade<br>3. 4th grade<br>4. 5th grade<br>5. 6th grade<br>6. 7th grade<br>7. 8th grade<br>8. 9th grade<br>9. 10th grade<br>10. 11th grade<br>11. 12th grade<br>12. Didn't finish higher education<br>13. Finished higher education other than university<br>14. Didn't finish university<br>15. Graduated from a university<br>16. Adult education<br>17. Other basic education<br>18. Other church/mosque education<br>30. Other	
Now I would like to talk to you about some of the characteristics of the household you lived in growing up			
113	What type of material was used for the roof of this house immediately before you moved?<br><br>IF THEY DON'T KNOW PROBE OR ASK THEM TO DESCRIBE IT	THATCH/LEAF…………………………1<br>RUSTIC MAT/PLASTIC SHEETS............2<br>WOOK PLANK………………………….3<br>CORRUGATED IRON…………………4<br>CALAMINE/CEMET FIBER………….5<br>OTHER (SPECIFY)…………………….6	
114	What material was used for the walls of that house at the time you moved?<br><br>IF THEY DON'T KNOW PROBE OR ASK THEM TO DESCRIBE IT	MUD………………………………………1<br>MUD AND WOOD………………………2<br>MUD AND STONE………………………3<br>MUD, WOOD AND STONE…………….4<br>UNCOVERED ADOBE………………….5<br>COVERED ADOBE……………………...6<br>CEMENT…………………………………7<br>BRICKS…………………………………...8<br>STONE WITH LIME/CEMENT……….9<br>PLYWOOD……………………………...10<br>WOOD PLANKS/SHINGLES…………11<br>OTHER (SPECIFIY)……………………12<br>98.DK	
115	Did you share any toilet facility you used at that time with other households?	YES …………………………1<br>NO…………………………..2<br><br><br>NO TOILET FACILITIES….3	

		DONT KNOW…………….98	
116	How many individuals lived in your household most of the time since you were a child?	NUMBER ☐☐<br>DON'T REMEMBER…………………98	
117	How many rooms did your household have in total, including rooms for sleeping and all other rooms?	NUMBER ☐☐<br><br>DON'T REMEMBER…………………98	
118	How many rooms were used for sleeping in the household?	NUMBER OF ROOMS FOR SLEEPING ☐☐<br>DON'T REMEMBER…………………98	
119	Who in your household had the final word in decisions involving:<br><br>A.YOU AND YOUR BROTHER/SISTER'S EDUCATION	MOTHER…… 1<br>FATHER…… …… 2<br>BOTH MOTHER AND FATHER…. 3<br>ME OR SIBLINGS…… 4<br>OTHER HOUSEHOLD MEMBERS….. 5<br>OTHER (SPECIFY)……… 96	
	B. HOW YOUR FAMILY SPENDS MONEY ON FOOD	MOTHER…… 1<br>FATHER…… …… 2<br>BOTH MOTHER AND FATHER…. 3<br>ME OR SIBLINGS…… 4<br>OTHER HOUSEHOLD MEMBERS….. 5<br>OTHER (SPECIFY)……… 96	
	C. HOW YOUR FAMILY SPENDS MONEY ON CLOTHING	MOTHER…… 1<br>FATHER…… …… 2<br>BOTH MOTHER AND FATHER…. 3<br>ME OR SIBLINGS…… 4<br>OTHER HOUSEHOLD MEMBERS….. 5<br>OTHER (SPECIFY)……… 96	
	D. LARGE INVESTMENTS SUCH AS BUYING A HOUSE, CAR	MOTHER…… 1<br>FATHER…… …… 2<br>BOTH MOTHER AND FATHER…. 3<br>ME OR SIBLINGS…… 4<br>OTHER HOUSEHOLD MEMBERS….. 5<br>OTHER (SPECIFY)…… 96	
120	When you were growing up, was your mother employed for a wage, salary, commission, or payment of any kind?	ALWAYS……………………………….. 1<br>MOST OF THE TIME………………...…2<br>SOMETIMES……………………………3<br>NEVER………………………………….98.<br>Do not remember……………………… 99	→ 122

121	What was the usual type of employment your mother engaged in?	PROFESSIONAL/TECHNICAL/<br>MANAGERIAL ……………………..…1<br>CLERICAL……………………………2<br>SALES AND SERVICES………….........3<br>SKILLED MANUAL LABOR……........4<br>UNSKILLED MANUAL LABOR……..5<br>DOMESTIC SERVICE………………..6<br>AGRICULTURE………………………7<br>OTHER (SPECIFY)…………………...8<br>DON'T KNOW………………………98	
122	Do you have sisters (from the same mother) who are already married?	YES…………………………………….1<br>NO……………………………………..2	→ 201
123	What age(s) did your sister(s) marry at?<br><br>PROBE: Were there any others?	AGE SISTER 1 ☐<br><br>AGE SISTER 2 ☐<br><br>AGE SISTER 3 ☐	

Section 2. Marriage, family formation and relationship

Now I would like to ask about marriage and how it happens.

201	Were you ever been promised in marriage?	YES…………………………………………..1<br>NO…………………………………………..2<br>DON'T KNOW……………………………..98	 →206<br>→206
202	How old were you when you first heard that you were promised in marriage?<br><br>REPORT AGE IN YEARS	AGE IN COMPLETED YEARS ☐☐<br><br>UNSURE………………………………98	→204
203	IF ANSWERED 'UNSURE' IN 202, PROBE: How many years ago did you first hear that you were promised in marriage?	TIME IN COMPLETED YEARS ☐☐<br>DON'T KNOW…………………….. ..98	
204	At the time you heard that your had been promised in marriage, had you ever met that person?	YES…………………………………………..1<br>NO…………………………………………..2<br>DON'T KNOW……………………………..98	
205	Who made the decision about who you would be promised to in marriage?	MYSELF ALONE …………………… ……1<br>MYSELF AND MY PARENTS ……………..2<br>FATHER ALONE………………………… .3<br>MOTHER ALONE………………………… 4<br>FATHER AND MOTHER TOGETHER… .5<br>ANOTHER FAMILY MEMBER………… .6<br>OTHER(SPECIFY) …………………………7<br>DON'T KNOW……………………………98	
206	Are you currently married or living together with a man as if married?	NOT CURRENTLY IN COUPLE:…… ……0<br>YES CURRENTLY MARRIED………… ….1<br>YES LIVING WITH A MAN…………… ….2<br>DIVORCED/SEPARATED…………………3<br>WIDOW……………………………… …..4	→208<br><br><br>→208<br>→ 208

	FOR THOSE CURRENTLY MARRIED OR PARTNERED: USING HUSBAND'S NAME AND AGE, IDENTIFY WHO HE IS ON THE HOUSEHOLD ROSTER. POINT TO HIS NAME AND ASK "Is this your husband?" IF YES, NOTE DOWN THE ROSTER NUMBER.	ROSTER NUMBER OF HUSBAND ☐☐	
207	Is your CURRENT husband/partner living with you now or is he staying elsewhere?	LIVING WITH HER……………………..1<br>STAYING ELSEWHERE………………...2<br>RESPONSE…………………………….99	
208A.	How long have you been separated/divorced or widowed?	1. Three or less years<br>2. More than three years<br><br>**Note: If more than three years Q234- 244 should be skipped**	
208	Have you been married or lived with a man only once or more than once?	ONLY ONCE………………….1<br>MORE THAN ONCE…………2	215
209	In what month and year did you start living with your FIRST husband / partner?	MONTH --------☐☐<br><br>DON'T KNOW MONTH …………….98<br><br>YEAR ☐☐☐☐<br><br>DON'T KNOW YEAR …………..9899	
210	How old were you when you started living fulltime with the first man you were married with?	AGE IN COMPLETED YEARS ☐☐	
211	Did you have any kind of marriage ceremony to formalize the FIRST union? What type of ceremony did you have?<br><br>MARK ALL THAT APPLY	NONE……………………………………A<br>CIVIL MARRIAGE…………………… …B<br>RELIGIOUS MARRIAGE……………. .C<br>CUSTOMARY MARRIAGE………… .D<br>OTHER (SPECIFY)…………………….. X	
212)	Did you move to live permanently with your FIRST husband before your marriage ceremony, immediately after the ceremony, or more than two months after the ceremony?	BEFORE……………………………………1<br>IMMEDIATELY AFTER……………………2<br>MORE THAN TWO MONTHS AFTER……3	
213	Did your first marriage involve a dowry/bride price payment?	YES, DOWRY……..…………………..…..1<br>YES, BRIDE PRICE…………………..…..2<br>NO……………………………………..……3<br>DON'T KNOW……………………..……98	 <br>→215<br>→215
214	Has all of the dowry/bride price for your first marriage been paid or does some part still remain to be paid?	ALL PAID……………………………………1<br>PARTIALLY PAID…………………………..2<br>NONE PAID……………………..……..….3	

215	Now I would like to ask about when you started living with your CURRENT or MOST RECENT husband / partner. In what month and year was that?	Month ☐☐<br><br>Don't know month…………………………..…98<br>Year ☐☐☐☐<br><br>Don't know year…………………………..9898	
216	What is the first name and age of your current or most recent husband?	A.FIRST NAME ____________________<br><br>B.AGE IN COMPLETED YEARS ☐☐	
217	How old were you when you started living fulltime with your current/most recent husband/partner?	AGE IN COMPLETED YEARS ☐☐	
218	What kind of marriage ceremony, if any, did you have in your CURRENT/MOST RECENT union?<br><br>Mark all that apply.	NONE ………………………………………1<br>CIVIL MARRIAGE ……………………… 2<br>RELIGIOUS MARRIAGE ………………….3<br>CUSTOMARY MARRIAGE ……………… 4<br>OTHER (SPECIFY) ..……………………….96	→222
219	Had you met your CURRENT/MOST RECENT husband before your wedding ceremony?	Yes…………………………………………...1<br>No…………………………………………...2	 →222
220	Did you spend any time living temporarily with your CURRENT/MOST RECENT husband before your marriage ceremony?	YES…………………………………………..1<br>NO……………………………………………2	
221	Did you move to live permanently with your current/most recent husband before your marriage ceremony, a little while after the ceremony, or immediately after the ceremony?	BEFORE……………………………………1<br>IMMEDIATELY AFTER……………………2<br>MORE THAN TWO MONTHS<br>AFTER……………………………………….3	
222	Did your current/most recent marriage involve a dowry/bride price payment?	YES, DOWRY……..…………………………1<br>YES, BRIDE PRICE…………………………2<br>NO……………………………………………3<br>DON'T KNOW………………………………98	 <br>→224<br>→224
223	Has all of the dowry/bride price for your current/most recent marriage been paid for, or does some part still remain to be paid?	ALL PAID……………………………………1<br>PARTIALLY PAID…………………………..2<br>NONE PAID…………………………………3<br>Do not know…………………………………98	
224	Does your CURRENT/MOST RECENT husband/partner have other wives or does he live with other women as if married?	YES…………………………………………..1<br>NO………………………………………….. 2<br>Do not know ………………………………..98<br>NA………………………………………… 99	 →227<br>→227<br>→227
225	Including yourself, in total, how many wives or partners does/did your CURRENT/MOST RECENT	TOTAL NUMBER OF WIVES ☐☐<br><br>AND LIVE-IN PARTNERS<br>DON'T KNOW………98	

	husband/partner live with now as if married?		
226	Are you the first, second, … wife	Rank…………….	

227	Did you own any of these when you came to live with your CURRENT/MOST RECENT husband/ partner?		
	A. LAND	YES…………………………………………..1<br>NO……………………………………..……..2	
	B. HOUSE	YES…………………………………………..1<br>NO……………………………………..…..2	
	C. POULTRY	YES…………………………………………..1<br>NO……………………………………..…..2	
	D. GOATS, PIGS AND OTHER SMALL ANIMALS	YES…………………………………………..1<br>NO……………………………………..…..2	
	E. CATTLE	YES…………………………………………..1<br>NO……………………………………..…..2	
	F. JEWELERY	YES…………………………………………..1<br>NO……………………………………..…..2	
	G. CASH	YES…………………………………………..1<br>NO…………………………………………..2	
	H. None	YES…………………………………………..1<br>NO…………………………………………..2	

	I. Other (specify)		
228<br>(223)	Do you still own any of the following, by yourself or with family or friends other than your husband?<br><br>A LAND<br><br>B HOUSE<br><br>C POULTRY<br><br>D GOATS, PIGS AND OTHER SMALL ANIMALS<br><br>E CATTLE<br><br>F JEWELERY<br><br>G CASH<br>H. None<br><br>I. Other (specify)	 <br>YES……………………………………..1<br>NO……………………………………..2<br><br>YES……………………………………..1<br>NO……………………………………..2<br><br>YES……………………………………..1<br>NO……………………………………..2<br><br>YES……………………………………..1<br>NO……………………………………..2<br><br>YES……………………………………..1<br>NO……………………………………..2<br><br>YES……………………………………..1<br>NO……………………………………..2<br><br>YES……………………………………..1<br>NO……………………………………..2	
229	Before getting married (for the first time), were you asked whether you wanted to marry your husband?	YES……………………………………..1<br>NO……………………………………..2	
230	What are the advantages/benefits of marrying at a younger age? By younger, I mean at age 15 or 16 or earlier.<br><br>SELECT ALL MENTIONED	STRONG TIES BETWEEN FAMILIES…….A<br><br>PARENTS CAN SEE GRANDCHILDREN BEFORE PARENTS DIE………………..….B<br><br>PARENTS CAN SEE CHILDREN MARRIED BEFORE PARENTS DIE……………………..C	

		MARRIED BEFORE PARENTS DIE………..D<br>AVOID SOCIAL SHAME/STIGMA…….……. D<br>GET SERVICE OF FUTURE SON IN LAW…………………………………… ……E<br>AVOID ABDUCTION………… …… F<br>AVOID PREMARITAL SEX…… ……. G<br>ENSURE VIRGINITY AT MARRIAGE…. … .H<br>REDUCE VULNERABILITY TO HIV… …...I<br>DIFFICULT TO MARRY AT OLDER AGE…..J<br>NO ADVANTAGES/BENEFITS……………..K<br>DON'T KNOW……………………………….L<br>OTHER (SPECIFY)……………………………X	→232
231	Of the advantages you mentioned, which is the biggest or most important?<br>Write letter of response	LETTER ☐	
232	What are the disadvantages/cost of marrying at a younger age? By younger, I mean at age 15 or 16 or earlier.<br>CIRCLE ALL MENTIONED	GREATER POVERTY……………………A<br>LESS EDUCATION……………………….B<br>POORER HEALTH OF WOMNE……….C<br>POORER HEALTH OF CHILDREN…….D<br>LESS ABILITY TO MAKE OWN DECISIONS……E<br>NO DISADVANTAGE…………………F<br>DO NOT KNOW………………………..G<br>OTHER (SPECIFY)………………………H	→ 234
233	Of the disadvantages you mentioned, which is the biggest or most important?<br>Write letter of response	LETTER ☐	
Relationship with husband			
When two people marry or live together they usually share both good and bad moments. I would now like to ask you some questions about your current or most recent relationships and how your husband or partner treats (treated) you. If anyone interrupts us I will change the topic of conversation. I would like to assure you that the answers will be kept secret and that you do not have to answer any questions that you do not want to.			

234	Would say that at this time, your family life is very happy, reasonably happy, somewhat unhappy, very unhappy, or neither happy nor unhappy?	VERY HAPPY…………………………..1<br>REASONABLY<br>HAPPY……………………..2<br>NEITHER HAPPY NOR UNHAPPY……3	

		SOMEWHAT<br>UNHAPPY…………………..4<br>VERY<br>UNHAPPY……………………………5<br>REDUSED…………………..99	
235	In general, do(did) you and your (current or most recent) husband discuss the following things together, often, sometimes, or never?		
	A Things that happened to him during the day	OFFTEN…………………………………1<br>SOMETIMES……………………………2<br>NEVER…………………………………..3<br>REFUSED………………………………99	
	B Things that happened to you during the day	OFFTEN…………………………………1<br>SOMETIMES………………………… 2<br>NEVER…………………………………..3<br>REFUSED…………………………… .99	
	C Your worries or feelings	OFFTEN…………………………………..1<br>SOMETIMES……………………………2<br>NEVER…………………………………..3<br>REFUSED………………………………..99	
	D His worries or feelings	OFFTEN…………………………………..1<br>SOMETIMES……………………………2<br>NEVER…………………………………..3<br>REFUSED………………………………..99	
	E His hopes for the future	OFFTEN…………………………………..1<br>SOMETIMES……………………………2<br>NEVER…………………………………..3<br>REFUSED………………………………..99	
	F Your hopes for the future	OFFTEN…………………………………..1<br>SOMETIMES……………………………2<br>NEVER…………………………………..3<br>REFUSED………………………………..99	
	G The health needs of your children	OFFTEN…………………………………..1<br>SOMETIMES……………………………2<br>NEVER…………………………………..3<br>REFUSED………………………………..99	
	H What to spend money on	OFFTEN…………………………………..1<br>SOMETIMES……………………………2<br>NEVER…………………………………..3<br>REFUSED………………………………..99	

	I Things that happen in the community	OFTEN……………………………….1<br>SOMETIMES…………………………2<br>NEVER……………………………….3<br>REFUSED…..………………………...99	
	J The education of children	OFTEN……………………………….1<br>SOMETIMES…………………………2<br>NEVER……………………………….3<br>REFUSED…..………………………...99	
	K Birth spacing	OFTEN……………………………….1<br>SOMETIMES…………………………2<br>NEVER……………………………….3<br>REFUSED…..………………………...99	
236	I am now going to ask you about some situations that are true for many women. Thinking about your (current or most recent) husband/partner and in-laws, would you say it is generally true that he/they:<br><br>A. Tries(d) to keep you from seeing your friends?<br>B. Tries to restrict contact with your family of birth?<br>C. Insists on knowing where you are at all times?<br>D. Ignores you and treats you indifferently?<br>E. Gets angry if you speak with another man?<br>F. Is often suspicious that you are unfaithful?	FOR EACH CATEGORY, PLEASE USE THE FOLLOWING RESPONSE CATEGORIES:<br>YES……………………………………..1<br>NO……………………………………...2<br>REFUSED……………………………..99<br><br>Current/most recent husband | Current/most recent in-laws<br>A. ☐ ☐<br>B. ☐ ☐<br>C. ☐ ☐<br>D. ☐ ☐<br>E. ☐ ☐<br>F. ☐ ☐	
237	In the past 12 months, has your CURRENT/MOST RECENT husband/partner told you not to use any birth contror?	YES……………………………………..1<br>NO……………………………………..2<br>NOT IN RELATIONSHIP IN PAST 12 MONTHS……………………………….97	
238	In the past 12 months, has your current husband/partner hurt you physically because you did not agree to get pregnant?	YES……………………………………..1<br>NO……………………………………..2	

		NOT IN RELATIONSHIP IN PAST 12 MTHS………………………………… ..97	
239	In the past 12 months, has your current husband/partner taken your birth control (such as pills) away from you or kept you from going to the clinic to get birth control so that you would get pregnant?	YES………………………………………..1<br>NO………………………………………..2<br>NOT IN RELATIONSHIP IN PAST 12 MTHS………………………………..... 97	
240	In general, does your partner support your decision about when or if you want to become pregnant?	YES………………………………………..1<br>NO………………………………………..2<br>NOT IN RELATIONSHIP IN PAST 12 MTHS……………………………………97	

EXPERIENCE WITH VIOLENCE

I would now like to ask you some questions about your current and past relationships and how your husband or partner treats or treated you. If anyone interrupts us I will change the topic of conversation. I would like to assure you that the answers will be kept secret and that you do not have to answer any questions that you do not want to.

241	How many times has a current or previous husband or partner:	1.In the last 12 months	3. Before the last 12 months
A.<br><br>B	Insulted or made you feel bad about yourself?<br><br>Belittled or humiliated you in front of other people?	NEVER………………..1<br>ONCE…………………2<br>FEW………………… ...3<br>MANY…………………4<br>NA……………………97<br>REFUSED……………99	NEVER………………..1<br>ONCE…………………2<br>FEW………………… ...3<br>MANY…………………4<br>NA……………………97<br>REFUSED……………99
c.	Done things to scare or intimidate you on purpose? For example, by the way he looked at you, by yelling and smashing things).	NEVER………………..1<br>ONCE…………………2<br>FEW………………… ...3<br>MANY…………………4<br>NA……………………97<br>REFUSED……………99	NEVER………………..1<br>ONCE…………………2<br>FEW………………… ...3<br>MANY…………………4<br>NA……………………97

			REFUSED……………99
D	Threatened to hurt you or someone you care about?	NEVER………………..1<br>ONCE…………………2<br>FEW………………… ...3<br>MANY…………………4<br>NA……………………97<br>REFUSED……………99	NEVER………………..1<br>ONCE…………………2<br>FEW………………… ...3<br>MANY…………………4<br>NA……………………97<br>REFUSED……………99
D	Slapped you or thrown something at you which could heart you?	NEVER………………..1<br>ONCE…………………2<br>FEW………………… ...3<br>MANY…………………4<br>NA……………………97<br>REFUSED……………99	NEVER………………..1<br>ONCE…………………2<br>FEW………………… ...3<br>MANY…………………4<br>NA……………………97<br>REFUSED……………99
E	Pushed or shoved you Hit you with fist or with something else which can heart you?	NEVER………………..1<br>ONCE…………………2<br>FEW………………… ...3<br>MANY…………………4<br>NA……………………97<br>REFUSED……………99	NEVER………………..1<br>ONCE…………………2<br>FEW………………… ...3<br>MANY…………………4<br>NA……………………97<br>REFUSED……………99
F	Hits you with fist or something else which could hurt you?	NEVER………………..1<br>ONCE…………………2<br>FEW………………… ...3<br>MANY…………………4<br>NA……………………97<br>REFUSED……………99	NEVER………………..1<br>ONCE…………………2<br>FEW………………… ...3<br>MANY…………………4<br>NA……………………97<br>REFUSED……………99
G	Kicked, drag, beaten, chock or burnt you?	NEVER………………..1<br>ONCE…………………2<br>FEW………………… ...3<br>MANY…………………4<br>NA……………………97<br>REFUSED……………99	NEVER………………..1<br>ONCE…………………2<br>FEW………………… ...3<br>MANY…………………4<br>NA……………………97<br>REFUSED……………99
H	Threaten to use or actually used gun, knife, or any other weapon against you?	NEVER………………..1<br>ONCE…………………2<br>FEW………………… ...3<br>MANY…………………4<br>NA……………………97<br>REFUSED……………99	NEVER………………..1<br>ONCE…………………2<br>FEW………………… ...3<br>MANY…………………4<br>NA……………………97<br>REFUSED……………99
I	Physically forced you to have sex when you didn't want to?	NEVER………………..1<br>ONCE…………………2<br>FEW………………… ...3<br>MANY…………………4<br>NA……………………97	NEVER………………..1<br>ONCE…………………2<br>FEW………………… ...3<br>MANY…………………4<br>NA……………………97

J<br><br><br><br><br><br>K	How often have you had sex with current husband/ partner when you did not want to because you were afraid that he might become violent?<br><br>How often have you been forced by your current husband/ partner or any other partner to do something sexual that you did not want to do?	REFUSED……………99<br><br>NEVER………………..1<br>ONCE…………………2<br>FEW………………… ...3<br>MANY…………………4<br>NA……………………97<br>REFUSED……………99<br><br>NEVER………………..1<br>ONCE…………………2<br>FEW………………… ...3<br>MANY…………………4<br>NA……………………97<br>REFUSED……………99	REFUSED……………99<br><br>NEVER………………..1<br>ONCE…………………2<br>FEW………………… ...3<br>MANY…………………4<br>NA……………………97<br>REFUSED……………99
242	CHECK RESPONSES ABOVE HAS WOMAN EXPERIENCED VIOLENCE?	Atleast one . 1<br>Not a single . 2	

I would now like to learn more about the injuries that you experienced from any of your partner's behaviour. By injury I mean any form of physical harm, including cuts, sprains, burns, broken bones or broken teeth, or other things like this. Please try to think of the most recent time this happened.

243	Did you ever go to any of the following for help?	YES	NO	
	A. POLICE	1	2	
	B. HOSPITAL OR HEALTH CENTER	1	2	
	C. SOCIAL SERVICES	1	2	
	D. LEGAL ADVICE CENTER	1	2	
	E. COURT	1	2	
	F. SHELTER	1	2	
	G. LOCAL LEADER			

		1	2	
	H. WOMEN'S ORGANIZATION	1	2	
	I. PRIEST/ RELIGIOUS LEADER	1	2	
	J. ANYWHERE ELSE? (SPECIFY)	1	2	
	K. Did not go to any of them	1	2	

**Section 3. Physical health**			
I would now like to ask a few questions about your health and use of health services.			
301	Now I would like to ask about your health in the past 4 weeks.<br>How would you describe your ability to walk around? Would you say that you have no problems, very few problems, some problems, many problems, or that you are unable to walk at all?	NO PROBLEMS…………1<br>VERY FEW PROBLEMS………..…2<br>SOME PROBLEMS……………. .3<br>MANY PROBLEMS………………… 4<br>UNABLE TO WALK AT ALL………5	
302	In the past 4 weeks, did you consult a doctor or other professional or traditional health worker because you yourself were sick?	YES……………………………………1<br>NO……………………………………..2<br>DON'T KNOW………………………98<br>REFUSED………………………..…99	

Mental health			
Now I want to talk about your situation in your household and how you feel about yourself. If anyone interrupts us, I will change the topic of conversation. I would like to assure you that the answers will be kept secret and that you do not have to answer any questions that you do not want to.			
303	How have you been feeling in general during the past four weeks?	In excellent spirits………………………………5<br>In very good spirits…………………...………4<br>In good spirits mostly………………………..3<br>I have been up and down in spirits a lot… ……2<br>In low spirits mostly…………………………….1<br>In very low spirits………………………………0	
304	How often were you bothered by any illness, bodily disorders, aches and pains during the past four weeks?	Every day…………………………………..5<br>Almost every day……………………………..4<br>About half of the time………………………..3<br>Now and then but les than half the time………2<br>Rarely…………………………………….1<br>None of the time……………………………0	
305	Did you feel depressed during the past four weeks?	Yes, to the point that I felt like taking my life…..5<br>Yes, to the point that I did not care about anything ……………………………………………...4<br>Yes, very depressed almost everyday ……………3<br>Yes, quite depressed several times………………2<br>Yes, a little depressed now and then …………1<br>No, never felt depressed………………………0	
306	Have you been in firm control of your behaviour, thought, emotions or feelings during the past four weeks?	Yes, definitely so………………………………5<br>Yes, for the most part………………………….4<br>Generally so……………………………………3<br>Not to well……………………………………..2<br>No, I am somewhat disturbed…………………..1<br>No, and I am very disturbed……………………0	
307	Have you been bothered by nervousness or your "nerves" during the past four weeks?	Extremely so- to the point where I could not work or take care of things…………………………..5<br>Very much so…………………………………4<br>Quite a bit……………………………………..3<br>Some- enough to bother me…………………..2<br>A little………………………………………..1<br>Not at all………………………………………0	
308	How much energy, pep, or vitality did you have or feel during the past month?	Very full of energy………………………………5<br>Fairly energetic most of the time……………… 4<br>My energy level varied quite a bit………………. 3<br>Generally low in energy or pep……………… 2<br>Very low in energy or pep most of the time… ..1<br>No energy at all - I feel drained………………..0	
309	How much have you felt downhearted and blue during the past four weeks.	None of this time……………………………. 5<br>A little of the time……………………………. 4<br>Some of the time……………………………… 3<br>A good bit of the time……………………….. 2<br>Most of the time……………………………… 1<br>All of the time………………………………. 0	
310	Were you generally tense or did you feel any tension during the past four weeks?	Yes (extremely tense, most/ all the time)………..5<br>Yes – very tense most of the time………………4<br>Not generally tense, but did feel fairly tense several times……………………………………………3<br>I felt a little tense a few times………………… 2<br>My general tension level was quite low………… 1<br>I never felt tense or any tension at all………….. 0	

311	How happy, satisfied, or pleased have you been with your personal life during the past four weeks?	Extremely happy – Could not have been more satisfied or pleased……………………………..5<br>Very happy most of the time………………… 4<br>Generally satisfied, pleased…………………….3<br>Sometimes fairly happy, sometimes fairly unhappy……………………………………… 2<br>Generally dissatisfied or unhappy………….. 1<br>My general tension level was quite low very dissatisfied or unhappy most or all the time …..0	
312	Did you feel healthy enough to carry out the things you like to do or had to do during the past four weeks?	Yes – definitely so…………………………….. 5<br>For the most part……………………………… 4<br>Health problems limited me in some important ways…………………………………………… 3<br>I was only healthy enough to take care of myself.. 2<br>I needed some help in taking care of myself……. 1<br>I needed someone to help me with most or all of the things I had to do…………………………. 0	
313	Have you felt so sad, discouraged, hopeless, or had so many problems that you wondered if anything was worthwhile during the past four weeks?	Extremely so - to the point that I have just about given up……………………………………….. 5<br>Very much so …………………………….. 4<br>Quite a bit ………………………………….. 3<br>Some - enough to bother me…………………….2<br>A little bit……………………………………..1<br>Not at all……………………………………….. 0	
314	How much did you feel fresh and rested after sleeping during the past four weeks.	None of the time ……………………………. 5<br>A little of the time ………………………….. 4<br>Some of the time ……………………………….3<br>A good bit of the time ………………………….2<br>Most of the time ………………………………. 1<br>All of the time………………………………… 0	
315	Have you been concerned, worried, or had any fears about your health during the past four weeks?	Extremely so…………………………………..5<br>Very much so………………………………….4<br>Quite a bit………………………………………3<br>Some, but not a lot……………………………2<br>Practically never……………………………….1<br>Not at all……………………………………….0	
316	Have you had any reason to wonder if you were losing your mind, or losing control over the way you act, talk, think, feel or of your memory during the past four weeks?	Yes, very much so and I am very concerned …..5<br>Some and I am quite concerned………………..4<br>Some and I have been a little concerned………..3<br>Some – but not enough to be concerned or worried about………………………………….2<br>Only a little ……………………………………1<br>Not at all……………………………………..0	
317	My daily life was full of things that were interesting to me or losing control over the way you act,talk, think, feel or of your memory during the past four weeks.	All of the time…………………………………5<br>Most of the time………………………………4<br>A good bit of the time…………………………..3<br>Some of the time……………………………….2<br>A little of the time……………………………..1<br>None of the time………………………………..0	

318	Did you feel active, vigorous, or dull, sluggish during the past four weeks?	Very active, vigorous every day………………….5<br>Mostly active, vigorous ……………………… 4<br>Fairly active, vigorous …………………………...3<br>Fairly dull, sluggish …………………………… 2<br>Most dull, sluggish ……………………….. 1<br>Very dull, sluggish every day……………………..0	
319	Have you been anxious, worried, or upset during the past four weeks?	Extremely so – to the point of being sick or almost sick ……………………………………………5<br>Very much so……………………………… 4<br>Quite a bit………………………………… 3<br>Some – enough to bother me………………… 2<br>A little bit……………………………………1<br>Not at all……………………………………0	
320	How much did you feel emotionally stable and sure of myself during the past four weeks.	None of this time………………………………5<br>A little of the time……………………………..4<br>Some of the time……………………………3<br>A good bit of the time…………………………..2<br>Most of the time……………………………...1<br>All of the time………………………………0	
321	Did you feel relaxed, at ease or high strung, tight, or keyed-up during the past four weeks?	Felt relaxed and at ease the whole month……..5<br>Felt relaxed and at ease most of the time……….4<br>Generally felt relaxed but at times felt fairly high strung…………………………………….3<br>Generally felt high strung but at times felt fairly relaxed……………………………...2<br>Felt high strung, tight, or keyed-up most of the time………………………………1<br>Felt high strung, tight, or keyed-up the whole month……………………………..0	
322	I felt cheerful, light hearted during the past four weeks.	None of this time……………………………..5<br>A little of the time……………………………..4<br>Some of the time………………………………3<br>A good bit of the time…………………………2<br>Most of the time………………………………..1<br>All of the time…………………………………0	
323	I felt tired, worn out, used up, or exhausted during the past four weeks.	None of this time……………………………..5<br>A little of the time……………………………..4<br>Some of the time………………………………3<br>A good bit of the time…………………………2<br>Most of the time………………………………..1<br>All of the time…………………………………0	
324	Have you been under or felt you were under any strain,stress, or pressure during the past four weeks?	Yes – almost more than I could bear or stand or stand ………………………………………….. 5<br>Yes – quite a bit of pressure……………………..4<br>Yes, some – more than usual…………………….3<br>Yes, some – but about usual……………………..2<br>Yes – a little…………………………………… 1<br>Not at all……………………………………..... 0	
325	Just now we talked about problems that may have bothered you in the past 4 weeks. I	YES………………………………………….. 1<br>NO………………………………………….. 2<br>REFUSED……………………………… 99	

	would like to ask you if, at any time in your life, you have ever thought about ending your life?		
326	Have you ever, at any time in your life, tried to take your life?	YES………………………………………….. 1<br>NO………………………………………….. 2<br>REFUSED……………………………… 99	
**Sexual and reproductive history**			
CHECK FOR THE PRESENCE OF OTHERS. BEFORE CONTINUING, MAKE EVERY EFFORT TO ENSURE PRIVACY.			
327	How old were you when you first menstruated?	NEVER MENSTRATED…………………0<br><br>AGE IN YEARS ☐☐	→329
328	How often did you miss school because of menstruation?	NEVER……………………………………….1<br>OCCASIONALLY…………………………...2<br>EVERY MONTH…………………………….3<br>WAS NOT AT SCHOOL AT TAHT AGE.........4<br>REFUSED…………………………………….99	
329	In a number of countries, there is a practice in which a girl may have part of her genitals cut ("Girizat"). Have you yourself ever had your genitals cut?	YES…………………………………………..1<br>NO…………………………………………..2<br>DK…………………………………………….98<br>REFUSED…………………………………99	
Now I would like to ask you some questions about sexual activity in order to gain a better understanding of some important life issues.			
330	How old were you when you had sexual intercourse for the very first time?	NEVER HAD SEXUAL INTERCOURSE….0<br><br>AGES IN YEARS ☐☐<br><br>FIRST TIME WHEN STARTED LIVING WITH (FIRST) HUSBAND/PARTNER….95	→401
331	When you first had sexual intercourse, did you want it then or would you have preferred to wait?	WAITED THEN…………………………….1<br>PREFERRED TO WAIT……………………2<br>NO<br>PREFERENCE………………………….3<br>REFUSED……………………………………<br>99	
332	Who decided that is was time for you to start having sex?	SELF ALONE……………………………….1<br>SELF WITH PARTNER…………………….2<br>PARTNER ALONE………………………....3<br>PARTNER WITH FAMILY………………....4<br>PARTNER'S FAMILY/PARENTS………….5<br>SOMEONE ELSE…………………………...6<br>REFUSED…………………………………..9<br>9	
333	When you first had sexual intercourse, did the other	YES…………………………………………..1<br>NO…………………………………………..2	

	person ask you if you wanted to have sex then?	REFUSED……………………………………99	
334	How frequently did you have sex with your partner in past month?	NOT AT ALL ………………………………..1<br>ONCE A MONTH………………………… 2<br>2-3 TIMES A MONTH………………………3<br>WEEKLY……………………………………4<br>2-3 PER WEEK…………………………… ..5<br>4+ PER WEEK…………………………… 6<br>REFUSED………………………………… 99	→336
335	Would you prefer that it (sex) be more or less frequent?	NO PREFERENCE…………………………0<br>LESS FREQENT………………………………..1<br>MORE FREQUENT……………………………..2<br>REFUSED……………………………….99	
336	Have you ever used anything or tried in any way to delay or avoid getting pregnant?	YES…………………………………………..1<br>NO……………………………………………..2	→341
337	How old were you when you first deliberately used something or tried in any way to avoid a pregnancy?	AGE ☐☐<br>DON'T REMEMBER…………………..98	
338	How many living children did you have at that time, if any?<br><br>IF NONE, RECORD '00'.	NUMBER ☐☐<br>DON'T REMEMBER…………………..98<br>REFUSED 99	
339	What was the first method you used?	FEMALE STERLIZATION……………..1<br>MALE STERLIZATION………………….2<br>PILL………………………………………3<br>IUD……………………………………...4<br>IMPLANTS…………………………………5<br>INJECTABLES……………………………..6<br>CONDOM…………………………………7<br>DIAPHRAGM/FOAM/JELLY……………8<br>RHYTHM METHOD……………………...9<br>WITHDRAWAL…………………………..10<br>PROLONGED BREASTFEEDING…….11<br>OTHER (SPECIFY)……………………….66	
340	Are you (or your spouse/partner) currently using a contraceptive method to avoid becoming pregnant?	YES…………………………………………..1<br>NO……………………………………………..2<br>DON'T KNOW…………………………98<br>NOT APPLICABLE………………………97	→342<br><br>→346<br>→346
341	Why are you currently not using a contraceptive method?	CURRENTLY PREGNANT/ BREAST FEEDING ………………………1<br>WANT TO BECOME PREGNANT ……2<br>INFREQUENT SEX/HUSBAND AWAY…3	All answers skip to 348

		HUSBAND/PARTNER<br>DISSAPROVES……..4<br>FEAR OF SIDE EFFECT …………….5<br>INCONVINIENT TO PROCURE/<br>UNAVAILABLE……………………6<br>HEALTH CONCERNS ……………7<br>DIFFICULT TO GET PREGNANT/<br>MENOPAUSAL …………………8<br>FATALISTIC ………………9<br>RELIGION DISSAPROVES ……10<br>DON'T KNOW ……………98<br>NO RESPONSE …………..99<br>Other……………………………………..97	
342	What contraceptive method are you using?	FEMALE STERLIZATION…………… …1<br>MALE STERLIZATION……………… ….2<br>PILL…………………………………… …..3<br>IUD………………………………………4<br>IMPLANTS………………………………5<br>INJECTABLES…………………………..6<br>CONDOM………………………………7<br>DIAPHRAGM/FOAM/JELLY…… …..8<br>RHYTHM METHOD………………… ….9<br>WITHDRAWAL…………………………..10<br>PROLONGED BREASTFEEDING…….11<br>OTHER (SPECIFY)………………………66	
343	For how long you have been using (CURRENT METHOD) now without stopping?	MONTH<br>DO NOT KNOW THE MONTH…..98<br><br>YEAR ☐☐☐☐<br><br>DO NOT KNOW THE YEAR…..9898	
342	Does your husband/partner know that you are using a method of family planning?	YES …………………………………..1<br>NO …………………………………2<br>DON'T KNOW…………………….3<br>NOT APPLICABLE ………………..4	
345	How involved are you in the decision about adopting a contraceptive method?	Not involved…………………………………1<br><br>Some input but final decision made by spouse..2<br><br>Jointly decide with spouse…………………... 3<br><br>I made final decision ………………………...4<br><br>Other household members decided…………5<br><br>Jointly decide with other household members...6<br><br>Medical provider decided ……………………7<br><br>Only one method available …………………..8	

		NOT APPLICABLE……………………….97	

346	Do you know of a place to obtain a contraceptive method?	YES…………………………1<br>NO………………………….2	
347	Can you obtain the contraceptive method of your choice?	YES…………………………1<br>NO…………………………2	
348	How involved are you in the decision about the number and spacing of children?	Not involved………………………1<br><br>Some input but final decision made by spouse..2<br><br>Jointly decide with spouse…………3<br><br>I made final decision ……………4<br><br>Other household members decided…5<br><br>Jointly decide with other household members...6<br><br>NOT APPLICABLE…………………97	
349	Have you ever been pregnant?	YES………………………………1<br>NO……………………………….2	→401
350	How many times have you ever been pregnant (including this time if you are currently pregnant)?	PREGNANCIES ☐☐	
351	Has a current or previous husband or boyfriend hurt you physically while you were pregnant?	YES……………………………..1<br>NO……………………………..2	

Now I will ask you about antenatal care, which refers to special health care that pregnant woman receive during their pregnancy.

352	Have you heard of antenatal care?	YES……………………………………..1<br>NO……………………………………..2	→354
353	How many times should a pregnant woman go to a health professional for antenatal care during her pregnancy?	ONE………………………………………1<br>TWO……………………………………. 2<br>THREE………………………………… 3<br>FOUR…………………………………….4<br>MORE THAN FOUR………………… .5<br>DON'T KNOW………………………… 98<br>REFUSED…………………………………99	
354	Now I would like to ask about all the births you have had during your life. Have you ever given birth?	YES……………………………………..1<br>NO……………………………………..2	→401
355	Do you have any sons or daughters to whom you have given birth who are now living with you? Continue	YES……………………………………..1<br>NO……………………………………..2	→357
357	Do you have any sons or daughters to whom you have given birth who are alive but do not live with you?	YES……………………………………..1<br>NO……………………………………..2	→359
358	How many sons are alive but do not live with you?	A.SONS ELSEWHERE ☐☐	

	And how many daughters are alive but do not live with you?<br><br>IF NONE, RECORD '00'.	B.DAUGHTERS ELSEWHERE	
359	Have you ever given birth to a boy or girl who was born alive but later died?<br>IF NO, PROBE: Any baby who cried or showed signs of life but did not survive?	YES……………………………………………..1<br>NO……………………………………………..2	 →361
360	How many boys were born alive but later died?<br><br>And how many girls were born alive but later died?<br><br>IF NONE, RECORD '00'.	BOYS DEAD<br><br><br>GIRLS DEAD	
	SUM ANSWERS TO 356, 358, AND 360, AND ENTER TOTAL.<br>IF NONE, RECORD '00'.	TOTAL	
361	CHECK 356, 358 and 360<br>Just to make sure that I have this right: you have had in TOTAL ___ births during your life. Is that correct?<br><br>YES ☐ NO ☐ → PROBE AND CORRECT THE NUMBER AS NECESSARY		
Employment History			

**Section 4. Employment**			
Now I would like to ask you some questions about any work you may done in the past before you were married.			
401	Did you ever work before you<br><br>Now I would like to ask you some questions about any work you may done in the past before you were married<br><br>Did you ever work before you were first married/were first married/ first lived with a man?<br><br>PROBE: Anything other than your usual housework?	YES………………………………..1<br><br>Yes ……………………………1<br><br>NO………………………………..2	→405
402	What was your last occupation before marriage/living with a man that is what work did you mainly do?	**1=Managers**<br>11=Chief Executives, Senior Officials and Legislators<br>12=Administrative and Commercial Managers<br>13=Production and Specialized Services Managers<br>14=Hospitality, Retail and Other Services Managers<br><br>**2=Professionals**<br>21=Science and Engineering Professionals<br>22=Health Professionals<br>23=Teaching Professionals<br>24=Business and Administrative Professionals<br>25=Information and Communication Technology Professionals<br>26=Legal, Social and Cultural Professionals<br><br>**3=Technicians and Associate Professionals**<br>31=Science and Engineering Professionals<br>32=Health Associate Professionals<br>33=Business and Administrative Associate Professionals<br>34=Legal, Social, Cultural and Related Associate<br>Professionals<br>35=Information and Communication Technicians<br><br>**4=Clerical Support Workers**<br>41=General and Keyboard Clerks<br>42=Customer Service Clerks<br>43=Numerical and Materials Recording Clerks<br>44=Other Clerical Support Workers<br><br>**5=Services and Sales Workers**<br>51=Personal Services Workers	

		52=Sales Workers<br>53=Personal Care workers<br>54=Protective Service Workers<br><br>**6=Skilled Agricultural, Forestry and Fishery Workers**<br>61=Market-oriented Skilled Agricultural Workers<br>62=Market-oriented Skilled Forestry, Fishery and Hunting Workers<br>63=Subsistence Farmers, Fishers, Hunters and Gatherers<br>**7=Craft and Related Trades Workers**<br>71=Building and Related Trades Workers (excluding Electricians)<br>72=Metal, Machinery and Related Trades Workers<br>73=Handicraft and Printing Workers<br>74=Electrical and Electronic Trades Workers<br>75=Food Processing, Woodworking, Garment and other<br>75=Craft and Related Trades Workers<br>**8=Plant and Machine Operators and** Assemblers<br>81=Stationary Plant and Machine Operators<br>82=Assemblers<br>83=Drivers and Mobile Plant Operators<br><br>**9=Elementary Occupations**<br>91=Cleaners and Helpers<br>92=Agricultural, Forestry and Fishery Labourers<br>93=Labourers in Mining, Construction, Manufacturing and Transport<br>94=Food Preparation Assistants<br>95=Street and Related Sales and Services Workers<br>96=Refuse Workers and Other Elementary Workers<br>10.Others	
403	Were you paid (in cash or in kind) for this work or were you not paid at all?	CASH ONLY…………………………..1<br>BOTH CASH AND KIND……………..2<br>IN KIND ONLY…………………………3<br>NOT PAID………………………………4	 <br>→405<br>→405
404	At that time who mainly decided how the money you earned would be used?	RESPONDENT ONLY………………1<br>PARENT(S)……………………………2<br>RESPONDENT WITH PARENT(S)……3<br>RESPONDENT WITH SOMEONE ELSE……………………4<br>SOMEONE ELSE……………………5<br>OTHER (SPECIFY)……………………96	

Now I would like to ask you some questions about any work you currently do			
405	Are you currently working?	YES………………………………….1<br>NO……………………………..2	 →407
406	Is your current job the same one that you just reported to me about?	YES……………………………..1<br>NO……………………………..2	→409<br>→408
407	At any time over the past 12 months, were you employed, including casual/part-time labour, for a wage, salary, commission OR any payment in kind, excluding temporary work, for anyone who is not a member of your household?	YES…………………………………………..1<br><br>NO…………………………………………..2	 <br>→417
408	Describe the main job over the past 12 months?<br><br>(	1=Managers<br>11=Chief Executives, Senior Officials and Legislators<br>12=Administrative and Commercial Managers<br>13=Production and Specialized Services Managers<br>14=Hospitality, Retail and Other Services Managers<br><br>2=Professionals<br>21=Science and Engineering Professionals<br>22=Health Professionals<br>23=Teaching Professionals<br>24=Business and Administrative Professionals<br>25=Information and Communication Technology Professionals<br>26=Legal, Social and Cultural Professionals<br><br>3=Technicians and Associate Professionals<br>31=Science and Engineering Professionals<br>32=Health Associate Professionals<br>33=Business and Administrative Associate Professionals<br>34=Legal, Social, Cultural and Related Associate Professionals<br>35=Information and Communication Technicians<br><br>4=Clerical Support Workers<br>41=General and Keyboard Clerks<br>42=Customer Service Clerks<br>43=Numerical and Materials Recording Clerks<br>44=Other Clerical Support Workers<br><br>5=Services and Sales Workers	

		51=Personal Services Workers<br>52=Sales Workers<br>53=Personal Care workers<br>54=Protective Service Workers<br><br>6=Skilled Agricultural, Forestry and Fishery Workers<br>61=Market-oriented Skilled Agricultural Workers<br>62=Market-oriented Skilled Forestry, Fishery and Hunting Workers<br>63=Subsistence Farmers, Fishers, Hunters and Gatherers<br><br>7=Craft and Related Trades Workers<br>71=Building and Related Trades Workers (excluding Electricians)<br>72=Metal, Machinery and Related Trades Workers<br>73=Handicraft and Printing Workers<br>74=Electrical and Electronic Trades Workers<br>75=Food Processing, Woodworking, Garment and other<br>75=Craft and Related Trades Workers<br><br>8=Plant and Machine Operators and Assemblers<br>81=Stationary Plant and Machine Operators<br>82=Assemblers<br>83=Drivers and Mobile Plant Operators<br><br>9=Elementary Occupations<br>91=Cleaners and Helpers<br>92=Agricultural, Forestry and Fishery Labourers<br>93=Labourers in Mining, Construction, Manufacturing and Transport<br>94=Food Preparation Assistants<br>95=Street and Related Sales and Services Workers<br>96=Refuse Workers and Other Elementary Workers	
409	Was this job working for the government or for a private company/person?	GOVERNMENT …… …1<br>PRIVATE COMPANY/PERSON……2	
410	Over the past 12 months, approximately how many months did your work at the main job?	NUMBER OF MONTHS ☐☐	
411	During these months, approximately how many weeks per month did you work at this main job?	NUMBER OF WEEKS ☐☐	

412	During these weeks, approximately how many days per week did you work at this main job?	NUMBER OF DAYS ☐☐	
413	How much was your last payment for wages/salary for the main job?	BIRR ☐	
414	What period of time do each of your salary/wages payments cover?	**TIME UNIT**<br>HOUR……………………………………..1<br>DAY…………………………………………..2<br>WEEK………………………………………..3<br>FORTNIGHT………………………………4<br>MONTH……………………………………….5<br>QUARTER……………………………………6<br>½<br>YEAR………………………………………..7<br>YEAR………………………………………...8	
415	If you were paid in kind the last time for your main job, approximately how much was your payment worth in birr (if, for example, you sold the goods to someone else)?	NOT PAID IN KIND………………………0<br><br>BIRR ☐	
416	How much do you usually receive in allowances or gratuities, including in-kind payments such as uniform, housing, food, and transport not included in the salary you just reported?<br><br>ESTIMATE CASH VALUE OF ANY IN-KIND PAYMENTS. WRITE "0" IF NONE.	BIRR ☐	
417	In the last 12 months, have you owned or co-owned with someone else a business enterprise?	YES…………………………………………..1<br>NO……………………………………………..2	 →425
418	Who if anybody do/did you own the business with?	SELF……………………………………1<br>FAMILY MEMBER(S)……………….2<br>WITH NON-FAMIY……………………3	
419	Which one of these best describe the type of the enterprise that you own or co-own?	FOOD PROCESSING/SALES……………..1<br>TAILOR SHOEMAKER…………………….2<br>HAIRDRESSER……………………………..3<br>FURNITURE MAKER/ELECTRICIAN….4<br>SELF EMPLOYED SKILLED (HEALTH PROVIDER TRANSLATOR, PARALEGAL, LAWYER)…………………………………...5	

		TAXI DRIVER……………………………….6<br>RESTAURANT / BAR OWNER…………..7<br>OTHER (SPECIFY)……………………….8	
420	Where did you receive the funds to start this enterprise?<br><br>MARK ALL THAT APPLY	AGRICULTURAL INCOME………………1<br>NON AGRICULTURAL INCOME………2<br>SALARY……………………………………3<br>SELLING ASSETS…………………………4<br>INHERITANCE……………………………5<br>BANK LOAN………………………………..6<br>MICROFINANCE……………………….7<br>OTHER (SPECIFY)……………………97	
421	Are the activities of the enterprise seasonal?	YES…………………………………………..1<br>NO…………………………………………..2	→423
422	How many months in the last 12 months was the enterprise active?	NUMBER __ __	
423	In the last 12 months what part of the household revenues were derived from this enterprise?	NONE…………………………..1<br>ABOUT 25%................................2<br>ABOUT HALF……………………3<br>ABOUT 75%..................................4<br>ALL……………………………………5	
424	Is the enterprise licensed?	YES…………………………………………..1<br>NO…………………………………………..2	
425	At any time over the past 12 months, did you work for other households, free of charge, as exchange labourer, or assist for nothing in return?	YES…………………………………………..1<br>NO…………………………………………..2	→427
426	Over the last 12 months, for how many times did you work for other households free of charge?	NUMBER ☐☐	
427	How many years in total have you worked in your life?	<2 YEARS...1<br>2-4 YEARS........................... ....2<br>5-9 YEARS...3<br>10 OR MORE YEARS.........................4<br>HAVE NEVER WORK………..5	
428	Do you have a bank account or an account in any other savings institution in your own name or jointly with someone else?<br><br>RECORD ALL MENTIONED	YES, IN OWN NAME…………………...A<br>JOINT ACCOUNT…………B<br>NO………………………………C	
429	Have you yourself ever taken out or been given a loan either in cash or in kind to start or expand a	YES…………………………………………..1<br>NO…………………………………………..2	

	business		
430	Do you own this or any other house either alone or jointly with someone else?	ALONE ONLY……………1<br>JOINTLY ONLY ………….2<br>DOES NOT OWN…………3<br>DKN………………………..4	432
431	Is your name on the title deed?	YES…………………………………………1<br>NO………………………………………..2<br>DOES NOT KNOW………….. .98	
431A	CHECK IF RESPONDENT HAS CHILDREN OF PRE-SCHOOL AGE (UNDER 6 YEARS) THEN ASK THE FOLLOWING Yes…..1 No……2		
432	Does any persons other than members of your family who are not residing with you help care for your children?	YES, MOST OF THE TIME…………….1<br>YES, SOMETIMES………………………..2<br>NO………………………………………..66	→ 434
433	How much do you pay for these services?	BIRR [ ]<br>DO NOT PAY THEM…..97	
CHECK QUESTIONS 206<br>IS CURRENTLY MARRIED OR LIVING WITH A PARTNER…………..1<br>CURRENTLY SEPERATED OR DIVORCED … ……. 2			→ 501
433A	Would you say that the money that you bring into the family is more than what your husband/partner contributes, less than what he contributes, or about the same as he contributes?	MORE THAN HUSBAND/PARTNER….1<br>LESS THAN HUSBAND/PARTNER……2<br>ABOUT THE SAME……………………...3<br>DO NOT KNOW…………………………98	
434	Have you ever given up/refused a job for money because your husband/partner did not want you to work?	Yes………………………………1<br>No……………………………….2	

435	Has your husband/partner ever taken your earnings or savings from you against your will?<br>IF YES: Has he done this once or twice, several times or many times?	ONCE OR TWICE…1<br>SEVERAL TIMES…..2<br>MANY TIMES/ ALL OF THE TIME..3	
436	Does your husband/ partner ever refuse to give you money for household expenses, even when he has money for other things?<br><br>A. IF YES: Has he done this once or twice, several	ONCE OR TWICE…1<br>SEVERAL TIMES…..2<br>MANY TIMES/ ALL OF THE TIME..3	
437	How involved are you in the decision about how the money you earn is used?	NOT INVOLVED…………………………0<br><br>SOME INPUT, BUT FINAL DECISION MADE BY SPOUSE……………………………….. ….... 1<br>JOINTLY DECIDED WITH SPOUSE ………..2<br>MAKE FINAL DECISION THEMSELVES………………………………….3<br><br>SOME OTHER HOUSEHOLD MEMBER… 4<br><br>JOINTLY DECIDES WITH OTHER HOUSEHOLD MEMBERS … 5<br><br>NA……………………………………..97	
438	Who usually decides how your (husband's/ partner's) earnings will be used: you, your (husband/partner), or you and your (husband/partner) jointly?	NOT INVOLVED…………………………0<br><br>SOME INPUT, BUT FINAL DECISION MADE BY SPOUSE……………………………….. ….... 1<br>JOINTLY DECIDED WITH SPOUSE ………..2<br>MAKE FINAL DECISION THEMSELVES………………………………….3<br>SOME OTHER HOUSEHOLD MEMBER… 4<br>JOINTLY DECIDES WITH OTHER HOUSEHOLD MEMBERS … 5<br>NA……………………………………..97	
